# Genome sequence data of *Streptomyces* sp. SS52, an endophytic strain for daidzein biosynthesis

**DOI:** 10.1016/j.dib.2019.104746

**Published:** 2019-11-04

**Authors:** Huong Van Nguyen, Phung Minh Truong, Huy Thuc Duong, Hiep Minh Dinh, Chuong Hoang Nguyen

**Affiliations:** a Center for Research and Application in Bioscience, Ho Chi Minh City, Viet Nam; b Ho Chi Minh City University of Education, Ho Chi Minh City, Viet Nam; c Agricultural Hi-Tech Park of Ho Chi Minh City, Ho Chi Minh City, Viet Nam; d University of Science, Vietnam National University Ho Chi Minh City, Ho Chi Minh City, Viet Nam

**Keywords:** Daidzein, *Streptomyces*, NGS, Genome sequence, SS52

## Abstract

We report here the biosynthesis of daidzein in *Streptomyces* sp. SS52, its genome sequence and the analysis of its genome for finding putative genes involved in daidzein biosynthesis. The *Streptomyces* sp. SS52 strain was isolated from the plant *Phyllanthus urinaria* in Tra Vinh province, Vietnam. This endophytic strain is capable of producing the isoflavone daidzein in the culture medium. *Streptomyces* sp. SS52 possesses a linear genome of 8,184,045 bp and the GC content of this genome is 72.5%. The preliminary genome analysis identified homologs of genes involved in the *de novo* biosynthesis of daidzein in the genome of *Streptomyces* sp. SS52. The genome sequencing of *Streptomyces* sp. SS52 was essential for the study of the biosynthesis of daidzein in *Streptomyces* bacteria.

**Table:** Specification Table

Subject	Biology
Specific subject area	Microbiology, Genomics, Biotechnology
Type of data	Table, complete genome sequence
How data were acquired	Chromatographic techniques, Nuclear Magnetic Resonance (NMR) spectroscopy, genome sequencing by PacBio Sequel and Illumina HiSeq 4000
Data format	Raw and Analyzed
Parameters for data collection	Isolation and identification of daidzein in the culture of *Streptomyces* sp. SS52 by chromatographic techniques and NMR spectroscopy. Genome sequencing of the strain by PacBio Sequel and Illumina HiSeq4000. Gene annotation and analysis by PGAP, ANI and BLAST programs.
Description of data collection	*Streptomyces* sp. SS52 was isolated from *Phyllathus urinaria* and cultured in the SS medium for daidzein production. Chromatographic techniques plus NMR spectroscopy were used to verify daidzein in the culture medium of the strain. Genomic DNA of *Streptomyces* sp. SS52 was extracted and sequenced by PacBio Sequel and HiSeq4000. Genome sequence was used for gene prediction and annotation as well as analysis to find putative genes involved in daidzein biosynthesis in *Streptomyces* sp. SS52.
Data source location	Center for Research and Application in Bioscience, Ho Chi Minh City, Vietnam
Data accessibility	Data are available within this article and the genome sequence of *Streptomyces* sp. SS52 is available in GenBank under the accession number NZ_CP039123.

**Table:** 

**Value of the Data** • *Streptomyces* sp. SS52 isolated from *Phyllathus urinaria* can produce daidzein, the isoflavone having several important biological activities. • The genome sequence data of *Streptomyces* sp. SS52 will be useful for the experimental identification of genes involved in the biosynthesis of daidzein. • In *Streptomyces* sp. SS52 genome, 7 putative genes involved in biosynthesis of daidzein were identified. • Data presented here could contribute to clarify the molecular mechanism of daidzein biosynthesis which is now poorly understood in *Streptomyces* bacteria.

## 1 Data

*Streptomyces* sp. SS52 was isolated from *Phyllanthus urinaria* in Tra Vinh province of Vietnam and this endophytic strain showed the capacity of producing daidzein in the culture medium. The presence of daidzein in the culture medium was confirmed by chromatographic techniques and NMR spectroscopy. Compound **1** was isolated from the culture of *Streptomyces* sp. SS52 as an amorphous powder. The ^1^H NMR spectrum of **1** revealed a 1,2,4-trisubstituted benzene ring including the aromatic protons at *δ*_H_ 7.96 (1H, d, *J* = 8.5 Hz), *δ*_H_ 6.96 (1H, dd, *J* = 8.5, 2.0 Hz) and 7.65 (1H, d, 2.0); a 1,4-disubstituted benzene ring assigned by two ortho-coupled protons at *δ*_H_ 7.37 (1H, d, *J* = 8.5 Hz) and *δ*_H_ 6.79 (1H, d, *J* = 8.5 Hz); a singlet olefinic proton at *δ*_H_ 8.28 and a hydroxy group at *δ*_H_ 9.51. The ^13^C NMR spectrum exhibited the presence of 13 carbon signals, consisting of one carbonyl carbon (*δ*_C_ 174.6), eight sp^2^ methines carbon, and five substituted sp^2^ carbons in the zone of 122–165 ppm (Table 1). These spectroscopic data were highly similar to those of daidzein [1], indicating that **1** was daidzein.

**Table 1:** The ^1^H NMR and^13^C NMR spectra of daidzein.

	1 (DMSO‑*d*_6_)	
	δ_Η_, J (Hz)	*δ*_C_
1		
2	8.28, br	152.9
3		104.1
4		174.6
5	7.96, d, 8.5	127.0
6	6.92, dd, 8.5, 2.0	115.0
7		162.3
8	6.86, d, 2.0	101.8
9		156.9
10		104.8
5-OH		
7-OH		
8-OH		
1’		122.6
2’/6’	7.37, d, 8.5	130.0
3’/5’	6.79, d, 8.5	114.9
4’		157.4
4′-OH	9.51, br	

*Streptomyces* sp. SS52 was selected for genome sequencing for identification of putative genes involved in the biosynthesis pathway of daidzein. The assembled genome of *Streptomyces* sp. SS52 has the size of 8,184,045 bp with the GC content of 72.5% and the coverage of 156-fold. The complete genome of *Streptomyces* sp. SS52 has the Average Nucleotide Identity (ANI) value of 99.98% with *Streptomyces* sp. CC71, the ANI value of 99.97% with *Streptomyces rochei* NRRL B-2410, the ANI value of 99.81% with *Streptomyces* sp. CCM_MD2014. As a result of gene prediction and annotation by the NCBI Prokaryotic Genome Annotation Pipeline, a total of 7320 genes was predicted including 6843 protein-coding genes, 67 tRNA genes, 3 ncRNA genes, and 18 rRNA (5S (6), 16S (6), 23S (6)) genes. In addition, a total of 389 pseudogenes was also predicted in the genome of *Streptomyces* sp. SS52 (Table 2).

**Table 2:** Features of the genome of *Streptomyces* sp. SS52.

Feature	*Streptomyces* sp. SS52
Source of isolation	*Phyllanthus urinaria*
Genome size (bp)	8,184,045
GC content (%)	72.5
Gene total	7320
Protein coding sequences	6843
tRNA	67
rRNA	6 (5S), 6 (16S), 6 (23S)
Pseudogenes	389

In plant, daidzein is synthesized by the phenylpropanoid pathway [2]. In this pathway, phenylalanine is first converted to cinnamate by phenylalanine ammonia lyase. Cinnamate is then transformed by cinnamate 4-hydroxylase to ρ-coumarate which is next converted to ρ-coumaroyl-CoA by 4-coumarate-CoA ligase. The ρ-coumaroyl-CoA starting unit is condensed by chalcone synthase and modified by chalcone reductase to give 4,2′,4′-trihydroxychalcone which is then converted to 7,4′-dihydroxyflavanone by chalcone isomerase. Finally, 7,4′-dihydroxyflavanone is converted to 7,4′-dihydroxyisoflavone (daidzein) by isoflavone synthase [3]. Homologous gene searching of the *Streptomyces* sp. SS52 genome using BLAST Program showed genes encoding proteins analogous to phenylalanine ammonia lyase, cinnamate 4-hydroxylase, 4-coumarate-CoA ligase, chalcone synthase, chalcone reductase, chalcone isomerase, isoflavone synthase in plants and in *Streptomyces clavuligerus* (Table 3).

**Table 3:** Putative genes involved in the biosynthesis of daidzein in the *Streptomyces* sp. SS52 genome.

Locus tag	Genome position	Annotated function	Analogous protein	Amino acid identity (%)	Functionally conserved amino acids (%)
E5N77_22775 (*hutH*)	4999095..5000633	Histidine ammonia-lyase	Phenylalanine ammonia-lyase (*Stylosanthes humilis*)	31	49
E5N77_23955	5262703..5264088	Cytochrome P450	Cinnamate 4-hydroxylase (*Glycine max*)	23	40
E5N77_15975	3550902..3552470 (complement)	4-coumarate-CoA ligase family protein	4-coumarate-CoA ligase (*Nicotiana tabacum*)	42	60
E5N77_05755	1315157..1316263	Type III polyketide synthase	Chalcone synthase (*Glycine max*)	28	45
E5N77_07535	1678955..1679926 (complement)	Aldo/keto reductase	Chalcone reductase (*Glycine max*)	35	54
E5N77_05760	1316260..1317474	Cytochrome P450	Cytochrome P450 (*Streptomyces clavuligerus*)	34	48
E5N77_23955	5262703..5264088	Cytochrome P450	Isoflavone synthase (*Glycine max*)	23	40

Phenylalanine ammonia lyase from the plant *Stylosanthes humilis* was used to search *Streptomyces* sp. SS52 genome and one matching protein, histidine ammonia lyase, was found. This 512 amino acid protein is encoded by *hutH* (locus tag E5N77_22775 in the *Streptomyces* sp. SS52 genome) and shares 31% amino acid identity (48% conserved residues) to the plant phenylalanine ammonia lyase for the whole protein sequence. Similarly, cinnamate 4-hydroxylase from *Glycine* max has an analogous protein in *Streptomyces* sp. SS52, cytochrome P450, with 24% identity (39% functionally conserved amino acids). This cytochrome P450 protein is encoded by a gene with the locus tag E5N77_23955 in the *Streptomyces* sp. SS52 genome. The highest scores in amino acid identity and functionally conserved amino acids were found between 4-coumarate-CoA ligase of *Nicotiana tabacum* and 4-coumarate-CoA ligase family protein of *Streptomyces* sp. SS52. The scores were 42% for amino acid identity and 60% for conserved amino acids. The 4-coumarate-CoA ligase family protein is encoded by a gene at the locus tag E5N77_15975 in the genome of *Streptomyces* sp. SS52. For the chalcone synthase searching, a single match corresponded to *Streptomyces* sp. SS52 type III polyketide synthase. This protein has 28% amino acid identity (45% conserved residues) to *G.* max chalcone synthase and is encoded by a gene with the locus tag E5N77_05755. Similarly, *G.* max chalcone reductase has an analogous protein in *Streptomyces* sp. SS52 which is aldo/keto reductase with 35% amino acid identity (54% conserved residues). The aldo/keto reductase is encoded by a gene with the locus tag E5N77_07535. Searching plant chalcone isomerase analog in *Streptomyces* sp. SS52 had no matching protein. Instead, a protein of *Streptomyces clavuligerus,* SCLAV_5491, with chalcone cyclization function was used to search for analogous protein in *Streptomyces* sp. SS52. SCLAV_5491 is a cytochrome P450 oxygenase and this protein was confirmed to be involved in naringenin biosynthesis in *S. clavuligerus* [4]. The protein analogous to SCLAV_5491 in *Streptomyces* sp. SS52 is also a cytochrome P450 which is encoded by a gene with the locus tag E5N77_05760. These two cytochrome P450 proteins of *S. clavuligerus* and *Streptomyces* sp. SS52 share 34% amino acid identity (48% conserved residues). Finally, using *G.* max isoflavone synthase for searching analogous protein in *Streptomyces* sp. SS52 resulted in a cytochrome P450 with 23% amino acid identity (40% conserved residues) for the whole protein. The cytochrome P450 of *Streptomyces* sp. SS52 is encoded by a gene with the locus tag E5N77_23955.

The genome sequence of *Streptomyces* sp. SS52 has been deposited in GenBank under the accession number NZ_CP039123.

## 2 Experimental design, materials and methods

For isolation and identification of daidzein, *Streptomyces* sp. SS52 was cultured in the SS agar medium [5] at 28 °C for 5 days. Then, the culture medium was extracted with ethyl acetate (EA). The solvent was evaporated under vacuum to obtain the EA extract. The EA extract was subsequently reextracted using solvents of increasing polarities: *n*-hexane, *n*-hexane-ethyl acetate 1:1, ethyl acetate to afford the corresponding extracts **H**, **HEA**, and **EA**. Extract **HEA** was applied to normal phase silica gel column chromatography (CC) and eluted with a solvent system of *n*-hexane−chloroform−ethyl acetate−acetone−acetic acid (isocratic, 350:100:40:25:10, v/v/v/v/v) to afford seven fractions: **HEA1**-**7**. Fraction **HEA4** was purified by CC with same solvent system as previously described to afford compound **1**.^1^H and ^13^C NMR spectra were acquired using Bruker AM-500 MHz spectrometer. Chemical shifts in ppm are referenced to the residual solvent signal (DMSO‑*d*_6_: *δ*_H_ = 2.50, *δ*_C_ = 39.5).

For genome sequencing using NGS technologies, *Streptomyces* sp. SS52 was cultured in Tryptic Soy Broth-containing baffled erlenmeyer at 28 °C. The erlenmeyer was shaken at 180 rpm for 3 days. The mycelium was harvested and washed with distilled water to remove the content of the medium before subjected to DNA extraction. Genomic DNA extraction was performed using the Qiagen MagAttract HMW kit (Qiagen) according to the instruction of the manufacturer. Library preparation and informatics was carried out by SNPSaurus (Eugene, OR). Genomic DNA was converted into sequencing libraries using the PacBio Multiplex kit and protocol (Pacific Biosciences, Menlo Park, CA) and sequenced with a PacBio Sequel using Sequencing Reagent Kit v2.1 by the University of Oregon GC3F facility. DNA was also converted to Illumina libraries using the Illumina Nextera DNA Flex kit (Illumina, San Diego, CA) and sequenced on a HiSeq4000 with paired-end 150 bp reads (Oregon GC3F facility). PacBio Sequel reads were assembled with Canu 1.7 [6] with a genome size of 8 Mbp and option corOutCoverage = 60. The Canu assembly was polished with the PacBio raw reads using the arrow program from PacBio. This consensus was then polished using Pilon [7] and the Illumina reads.

Pairwise average nucleotide identity (ANI) was performed for *Streptomyces* sp. SS52 and other *Streptomyces* strains in the database using Jspecies Web server [8]. Gene prediction and annotation were carried out using the NCBI Prokaryotic Genome Annotation Pipeline (http://www.ncbi.nlm.nih.gov/genome/annotation_prok). BLAST Program was used to search putative genes encoding protein analogous to the enzymes participating in the daidzein biosynthesis by the phenylpropanoid pathway.

## References

[1] Maskey R.P., Asolkar R.N., Speitling M., Hoffman V., Grun-Wollny I., Fleck W.F., Laatsch H. (2003). Flavones and new isoflavone derivatives from microorganisms: isolation and structure elucidation. Z. Naturforschung B.

[2] Gutierrez-Gonzalez J.J., Guttikonda S.K., Tran L.S., Aldrich D.L., Zhong R., Yu O., Nguyen H.T., Sleper D.A. (2010). Differential expression of isoflavone biosynthetic genes in soybean during water deficits. Plant Cell Physiol..

[3] Yu O., Jung W., Shi J., Croes R.A., Fader G.M., McGonigle B., Odell J.T. (2000). Production of the isoflavones genistein and daidzein in non-legume dicot and monocot tissues. Plant Physiol..

[4] Álvarez-Álvarez R., Botas A., Albillos S.M., Rumbero A., Martín J.F., Liras P. (2015). Molecular genetics of naringenin biosynthesis, a typical plant secondary metabolite produced by S*treptomyces clavuligerus*. Microb. Cell Factories.

[5] Sathish K.S., Kokati V.B. (2012). In-vitro antimicrobial activity of marine actinobacteria against multidrug resistance *Staphylococcus aureus*. Asian Pac. J. Trop. Biomed..

[6] Koren S., Walenz B.P., Berlin K., Miller J.R., Phillippy A.M. (2017). Canu: scalable and accurate long-read assembly via adaptive k-mer weighting and repeat separation. Genome Res..

[7] Walker B.J., Abeel T., Shea T., Priest M., Abouelliel A., Sakthikumar S., Cuomo C.A., Zeng Q., Wortman J., Young S.K., Earl A.M. (2014). Pilon: an integrated tool for comprehensive microbial variant detection and genome assembly improvement. PLoS One.

[8] Richter M., Rosselló-Móra R., Oliver Glöckner F., Peplies J. (2016). JSpeciesWS: a web server for prokaryotic species circumscription based on pairwise genome comparison. Bioinformatics.

